# Downregulation of Claudin5 promotes malignant progression and radioresistance through Beclin1-mediated autophagy in esophageal squamous cell carcinoma

**DOI:** 10.1186/s12967-023-04248-7

**Published:** 2023-06-11

**Authors:** Shan Huang, Jiayi Zhang, Yi Li, Yaqiong Xu, Hui Jia, Lei An, Xiaotan Wang, Yuting Yang

**Affiliations:** Department of Radiation Oncology, The Second Affiliated Hospital of Xi’an Jiaotong University, No. 157, Xi Wu Road, Xi’an, 710004 China

**Keywords:** Esophageal squamous cell carcinoma, Claudin5, Autophagy, Progression, Radioresistance

## Abstract

**Background:**

Esophageal squamous cell carcinoma (ESCC) is a highly prevalent and aggressive cancer with poor treatment outcomes. Despite the critical role of tight junction proteins in tumorigenesis, the involvement of Claudin5 in ESCC remains poorly understood. Thus, this study aimed to investigate the role of Claudin5 in ESCC malignant progression and radioresistance, as well as the underlying regulatory mechanisms.

**Methods:**

The expression of Claudin5 was evaluated in esophageal cancer tissue using both public databases and 123 clinical samples. CCK-8, transwell invasion, wound healing and clonogenic survival assays were used to examine the proliferation, invasion, migration and radiosensitivity of ESCC cells in vitro. Xenograft and animal lung metastasis experiments were conducted to examine the impact of Claudin5 on tumor growth and lung metastasis in vivo. The effect of Claudin5 on autophagy was detected via transmission electron microscopy, western blotting and autophagy flux. Immunohistochemical staining was used to detect Claudin5 expression in ESCC patient samples. The statistical difference was assessed with Student *t* test or one-way ANOVA. The correlation between Claudin5 expression and radiotherapy response rate was performed by the Chi-square test. The significance of Kaplan–Meier curves was evaluated by the Logrank test.

**Results:**

Claudin5 expression was downregulated in ESCC tissues. Downregulation of Claudin5 promoted ESCC cell proliferation, invasion, and migration both in vitro and in vivo. Downregulation of Claudin5 decreased the radiosensitivity of ESCC cells. Moreover, downregulation of Claudin5 promoted autophagy and the expression of Beclin1. Beclin1 knockdown reversed the effect of Claudin5 downregulation on autophagy induction and the promotion of ESCC cell malignant progression and radioresistance. Additionally, low expression of Claudin5 in ESCC cancer tissues was associated with poor radiotherapy response and prognosis.

**Conclusions:**

In summary, these findings suggest that downregulation of Claudin5 promotes ESCC malignant progression and radioresistance via Beclin1-autophagy activation and may serve as a promising biomarker for predicting radiotherapy response and patient outcome in ESCC.

**Supplementary Information:**

The online version contains supplementary material available at 10.1186/s12967-023-04248-7.

## Background

Esophageal cancer is a prevalent malignancy with squamous cell carcinoma and adenocarcinoma as its main pathological types [1]. In China, over 80% of esophageal cancer cases are esophageal squamous cell carcinoma (ESCC) [2]. Endoscopy and surgical resection are effective for early ESCC patients. About 70% of patients with esophageal squamous carcinoma are diagnosed at an advanced stage, largely because of the cells' high tendency for invasion and metastasis [2]. Radiotherapy is the primary treatment for advanced ESCC, but unfortunately, 40–60% of patients have poor responses to radiotherapy [3]. Thus, understanding the mechanism of tumor progression and treatment resistance in ESCC is essential.

Claudin5 (coded by *CLDN5* gene) is a member of the claudin protein family, which includes 27 integral membrane proteins [4]. Accumulated evidence suggests that claudin proteins are key regulator of carcinogenesis and metastasis [5]. Additionally, claudin proteins play a crucial role in cell signaling and are involved in numerous tumor-related signaling pathways that impact the progression of tumors [6, 7]. Previous studies have shown reduced Claudin5 expression in various cancers, yet its exact functions and downstream targets remain unclear.

Autophagy (macroautophagy or autophagy), a conservative catabolic process [8], has been found to play a significant role in cancer development [9, 10]. Beclin1, a protein with a BH3 domain, is phosphorylated by ULK1 and acts as a scaffold for the whole PI3K complex, facilitating the localization of autophagy proteins to phagocytic cells. ATG14 and UVRAG bind to Beclin1 to promote the interaction between Beclin1 and VPS34 and to promote phagocytosis formation [8]. Autophagy is a crucial mechanism for cell survival during survival stress [11–13], thereby promoting resistance of cancer cells to chemotherapy and radiotherapy [14–16]. Our previous study observed the activation of autophagy in radioresistance ESCC and showed that inhibiting autophagy improved radiosensitivity [17]. However, the role of Claudin5 in autophagy-mediated radioresistance remains unknown.

In this study, we investigated the role of Claudin5 in ESCC and demonstrated its potential as a prognostic indicator. We found that Claudin5 expression was suppressed in ESCC and that it played a vital role in inhibiting malignancy and radioresistance in ESCC cells. Our further mechanism research confirmed that Beclin1-induced autophagy mediated the effect of Claudin5 on ESCC cells. Clinical sample analysis revealed that Claudin5 expression was associated with both radiotherapy response and outcome in patients with ESCC, indicating its potential as a prognostic indicator.

## Methods

### Patients and clinical specimens

In this study, we recruited 123 primary ESCC patients between 2010 and 2011 at the First and Second Affiliated Hospital of Xi'an Jiaotong University. All patients was pathological diagnosis as esophageal squamous cell carcinoma. All patients were diagnosed at locally advanced stage according to AJCC Version 9 Cancer Staging System (https://www.facs.org/quality-programs/cancer-programs/american-joint-committee-on-cancer/version-9/). Patients underwent radical radiotherapy with a specific regimen consisting of 30 fractions administered at a dose of 2 Gy per fraction, given 5 fractions per week. To analyze the cohort, we used formalin-fixed, paraffin-embedded (FFPE) specimens, which were obtained by biopsy before radiotherapy. The clinical data of each patient was collected and sorted by two independent researchers. This study obtained ethics approval from the Medical Ethics Committee of Xi'an Jiaotong University and carried out in accordance with the provisions of the Helsinki Declaration of 1975. Informed consent for all patients has been obtained before the study.

Disease free survival (DFS) was defined from the date of diagnosis to the date of recurrence or death. Overall survival (OS) was defined from the date of diagnosis to the date of death for any reason. The efficacy of cancer radiotherapy was evaluated according to RECIST1.1 (https://recist.eortc.org/) by imaging examination systems and biopsy. Radiotherapy response included complete response (CR) and partial response (PR), and radiotherapy non-response included stable disease (SD) and progressive disease (PD).

### Cell culture, treatments, and transfection

The human ESCC cell lines TE1 and KYSE150 was obtained from the National Collection of Authenticated Cell Cultures. Cells were cultured in RPMI-1640 medium or DMEM/high sugar medium with 10% fetal bovine serum and maintained in a controlled environment at 37 °C and 5% CO_2_. To inhibit autophagy, cells were treated with 3-MA (5 mM, S2767; Selleck Chemicals, Houston, TX, USA) for 4 h.

The siRNA targeting Claudin5 and the Claudin5 overexpression vector were purchased from Tsingke Biotech Co., Ltd. (Beijing, China). The sh-Claudin5 lentivirus (H. sapiens), sh-Beclin1 lentivirus (H. sapiens), and their corresponding control lentiviruses were obtained from Tsingke Biotech Co., Ltd. (Beijing, China). Transient knockdown or overexpression of candidate genes was achieved by transfecting appropriate siRNA or gene overexpression plasmids. Transient transfection was performed using Lipofectamine 3000 (Invitrogen; Thermo Fisher Scientific, Waltham, MA, USA) according to the standard protocol. For viral infection, esophageal cancer cells were seeded in 48-well plates and grown to 50% confluence. The lentivirus (1 × 10^8^ TU/ml) was mixed with the infection reagent and added to the cells. After 48 h of infection, the culture medium was replaced, and puromycin (3 μg/ml) was added to the medium to select for successfully infected cells. The target sequences are presented in Additional file 1: Tables S1 and S2.

### Xenograft and lung metastasis assay

Four-week-old male BALB/c nude mice were obtained from the Animal Experiment Center at Xi'an Jiaotong University School of Medicine (Xi'an, China). TE1 cells (1 × 10^7^ cells) transfected with shRNAs targeting Claudin5 (sh-Claudin5) or corresponding negative control shRNAs (sh-NC) were subcutaneously injected into the axilla of each mouse. Tumor growth was monitored by measuring the length (a) and width (b) of the tumor every 3 days, and tumor volume was calculated using the formula V = ab^2/2. After 23 days, the mice were euthanized, and the tumors were harvested, weighed, and processed for further analysis. Additionally, the lungs were surgically excised and subjected to subsequent examination. The experimental procedures were performed in accordance with the guidelines and regulations approved by the Ethics Committee of the Health Science Center of Xi'an Jiaotong University.

### Western blotting

The western blotting experiments were carried out following previously described methods [17, 18]. Briefly, protein of ESCC cells was extracted using RIPA lysate (Thermo Fisher Scientific, Waltham, MA, USA) and separated by electrophoresis with 15% SDS-PAGE gel. Protein was then transferred to a PVDF membrane. After blocking, primary antibodies (anti-Claudin5, 1:1000, ab131259; anti-LC3, 1:1000, ab192890; anti-Beclin1, 1:1000, ab210498; Abcam, Cambridge, UK) were incubated with the membranes for 14 h. Then, secondary antibodies were used to incubate the membranes for 1 h at room temperature. The signals were detected with an ECL kit (Millipore, MA, USA), and analyzed by NIH-ImageJ software after final scans.

### qRT-PCR

The TRIzol reagent (Thermo Fisher Scientific, Waltham, MA, USA) was used to extract total RNA from ESCC cells. After RNA extraction, cDNA reverse transcription was performed using a PrimeScript™ RT reagent kit (TaKaRa, Japan). SYBR Premix Ex Taq™ II (TaKaRa, Japan) was then used to qRT-PCR. The internal reference used was GAPDH. The primer sequences used in this study are listed in Additional file 1: Table S3.

### Cell proliferation assay

The cell counting kit 8 assay (CCK8, Wanlei, Shenyang, China) assay was used to evaluate the proliferation ability of ESCC cells. First, the cells were seeded into a 96-well plate and cultured in a cell culture incubator until the detection time. According to the instructions of the kit, CCK-8 was added to the cell culture medium. Then, the absorbance of the cells at 450 nm was measured. The experiment was independently repeated three times. The mean and standard deviation were calculated and the results were plotted as a proliferation curve.

### Invasion assay

The cell invasion capacity was evaluated by transwell invasion assay. Prior to the experiment, the upper membrane was coated with 50 μl Matrigel (BD Biosciences, San Jose, CA, USA). Cells were resuspended in serum-free medium and then seeded into the upper chambers of a Transwell chamber (8 μm pore-size, Corning, NY, USA) at a density of 2 × 10^5^ cells. Next, medium containing 10% FBS was added to the lower chamber, and the cells were incubated in a cell culture incubator. After 24 h of incubation, the cells on the upper surface of the membrane were gently removed using a cotton swab. Cells that had invaded through the Transwell membrane were fixed with 4% paraformaldehyde and stained with 0.05% crystal violet. The stained cells were quantified under the view of microscope at 200 × magnification. The experiment was independently repeated three times.

### Migration assay

To assess the cell migration capacity, wound healing assay were conducted. Initially, ESCC cells were cultured in a 6-well plate until they adhered and reached 80% density. Subsequently, a scratch was created on the cell monolayer using a pipette tip and the medium was substituted with serum-free medium. The cells were allowed to incubate until the detection time. The scratch width was measured, and the experiment was repeated three times independently.

### Clonogenic survival assay to test cell radiosensitivity

To test the radiosensitivity of the cells, a clonogenic survival assay was performed using a method previously described [17, 18]. The cells were seeded into six-well plates at different densities, ranging from 100 to 7000 cells per well. They were then exposed to X-ray irradiation at various doses, ranging from 0 to 8 Gy, delivered in fractions of 2 Gy each. After irradiation, the cells were allowed to grow for 14 days. Then, the colonies containing at least 50 cells were counted using a light microscope. To visualize the colonies, a 0.1% solution of crystal violet was added. The surviving fraction of cells was calculated using the colony counts and normalized to the plating efficiency of the unirradiated cells.

The data were analyzed using a single-hit multitarget model (SF = 1 − (1 − e^ [− kD])^N) to estimate the sensitivity. All experiments were repeated three times. The survival curves were plotted using GraphPad Prism Version 9.5.1. Radiobiological parameters (*D*_0_, *D*_*q*_, *N*, and SF2) were calculated from the single-hit multitarget model (SF = 1 − (1 − e^ [− kD]) ^N). *D*_0_: final slope; *D*_*q*_: quasi-threshold dose; *N:* extrapolation number; SF2: survival fraction of 2 Gy.

### Transmission electron microscopy

The observation and quantification of autophagy with a double-layer membrane structure were conducted using transmission electron microscopy (TEM), following a previously described protocol [17]. To prepare for TEM, the cells were initially fixed in a specialized fixative solution (Electron Microscope Fixative from Servicebio Technology CO., LTD, Wuhan, China) and dehydrated using a series of acetone. Subsequently, the cells were embedded in a suitable medium, solidified, and cut into thin sections for TEM analysis by TEM HT7700 (Hitachi, Tokyo, Japan).

### Monitoring autophagy flux

The method for detecting autophagy flux was described in detail in our previous publication [17]. Its principle involves using mRFP-GFP-LC3 (Hanheng Biotechnology Co., Ltd., Shanghai, China) to label LC3. When lysosomes and autophagosomes fuse to form autolysosomes, the GFP fluorescence is quenched, while the mRFP fluorescence signal remains unchanged. Red dots represent autolysosomes. First, the cells need to be seeded in a confocal culture dish, and then mRFP-GFP-LC3 is transfected into ESCC cells for 6 h. LC3 puncta are observed using a laser confocal fluorescence microscope (IX83, Olympus, Tokyo, Japan). LC3 puncta were visualized using a laser confocal fluorescence microscope (IX83, Olympus, Tokyo, Japan) and analyzed using ImageJ software.

### Immunohistochemical staining

Our previous study described the detailed steps of immunohistochemical (IHC) staining [17]. FFPE specimens were cut into 4.5 μm thick slices. Then the slices were hydrated. After that, antigen retrieval was performed using citrate buffer solution. The tissue sections were blocked for 1 h and subjected to primary antibody incubation with anti-Claudin5 (1:1000, ab131259; Abcam, Cambridge, UK) overnight at 4 °C. The second day, secondary antibody labeled with HRP (1:1000, ab6721; Abcam, Cambridge, UK) were applied, and the samples were stained with DAB. IHC staining was assessed using the immunoreactive score (IRS) established by Remmele and Stegner [19] using Image-Pro Plus software. To compare Claudin5 protein expression between ESCC tissues and their corresponding adjacent normal tissues, we obtained adjacent normal tissue samples from 42 out of the 123 patient samples. These 42 normal adjacent tissue samples were used as a comparison group to evaluate the expression of Claudin5 in ESCC tissues. The specific calculation method of IRS was the same as our previous research [17]. The immunoreactive score (IRS) was calculated by combining the scores for staining intensity and the percentage of positive cells. The staining intensity was scored on a scale of 0–3, with 0 representing no color reaction, 1 indicating mild staining, 2 denoting moderate staining, and 3 representing intense staining. The percentage of positive cells was scored on a scale of 0–4, with 0 indicating no positive cells, 1 representing less than 10% positive cells, 2 indicating 10–50% positive cells, 3 representing 51–80% positive cells, and 4 representing more than 80% positive cells. The median value of IRS was employed to determine the cut-off. The level of Claudin 5 protein expression was identified as low-expression (IRS ≤ median) and high-expression (IRS > median).

### Statistical analysis

Statistical analysis was performed using GraphPad Prism Version 9.5.1 and SPSS 22 software. The normality of data distribution was assessed using the Shapiro–Wilk test. The data are presented as mean ± standard error of the mean (SEM) from at least three independent experiments. Statistical differences were evaluated using Student's *t*-test or one-way ANOVA, as appropriate. In cases where the data did not follow a normal distribution, the unpaired nonparametric Mann–Whitney U test was utilized, and the results were reported as the median. The correlation between Claudin5 expression and radiotherapy response rate was performed by the Chi-square test. The significance of Kaplan–Meier curves was evaluated by the Logrank test. All statistical tests were two-tailed, and a P-value below 0.05 was considered statistically significant.

## Results

### Downregulation of Claudin5 in ESCC

**Fig. 1:**
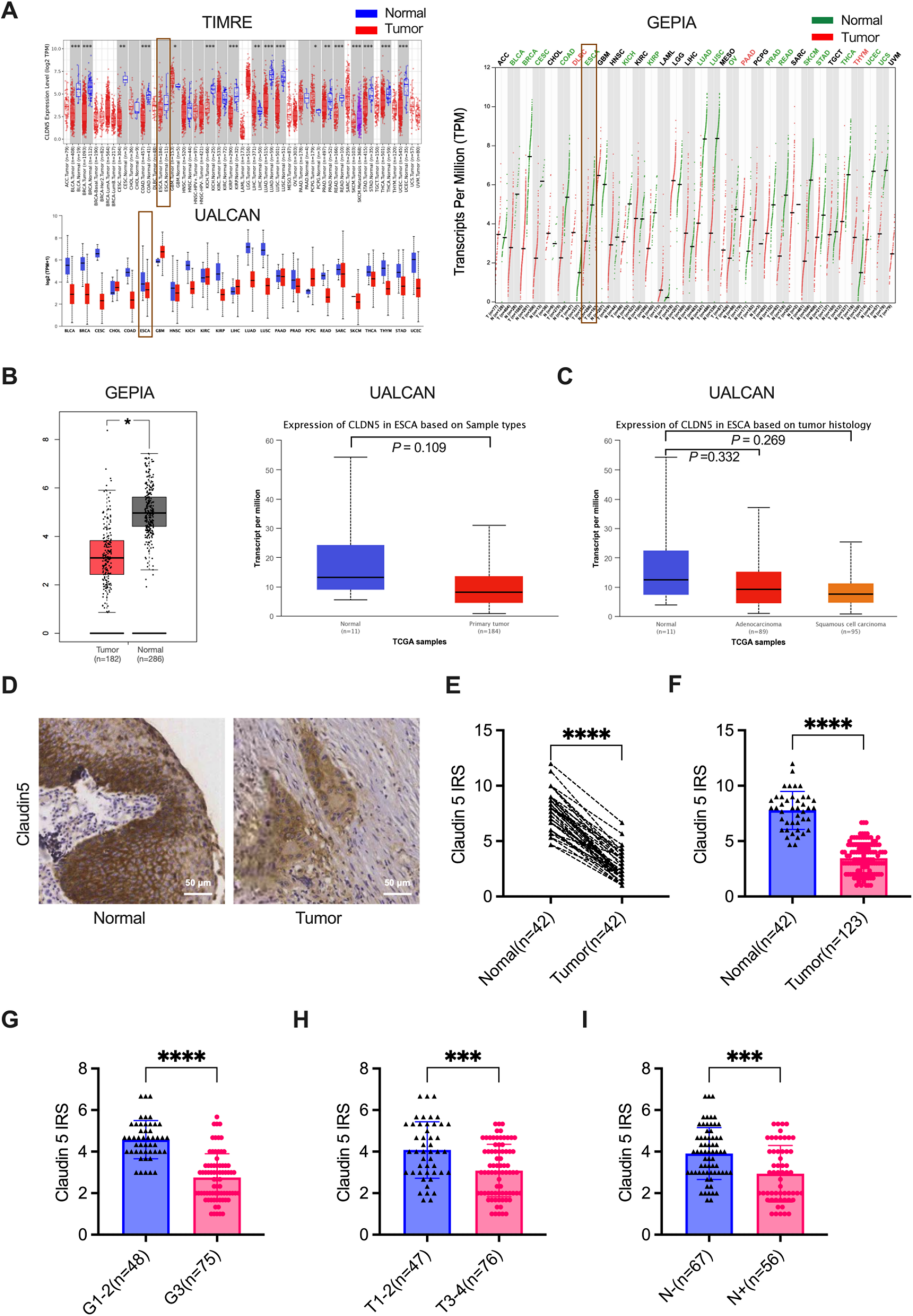
Reduced expression of Claudin5 in ESCC tissues and its Association with Tumor Progression. **A** *CLDN5* expression in pan-cancer using TIMER2.0 (http://timer.cistrome.org/), UALCAN (https://ualcan.path.uab.edu/), and GEPIA (http://gepia.cancer-pku.cn/) portals. **B**, **C** *CLDN5* expression in normal and esophageal carcinoma (ESCA) tissues using GEPIA and UALCAN portals. **D** Representative images of immunohistochemical (IHC) staining of Claudin5 protein in adjacent normal (n = 42) and tumor tissues (n = 123) from collected ESCC patients samples. **E** Immunoreactivity score (IRS) of Claudin5 in 42 ESCC tumor tissues and paired adjacent normal tissues. **F** IRS of Claudin5 protein in adjacent normal (n = 42) and tumor tissues (n = 123) from collected ESCC patients samples. **G** IRS of Claudin5 protein in ESCC samples between patients with grade 3 (G3) tumors and grades 1–2 (G1–2). **H** IRS of Claudin5 protein in ESCC samples between patients with T1–2 stage and T3–4. **I** IRS of Claudin5 protein in ESCC samples between patients with node metastasis and patients without. The statistical difference was assessed with the unpaired nonparametric Mann–Whitney U test in **E**,** F**,** G**, **H** and** I**. *p < 0.05, **p < 0.01, ***p < 0.001, and ****p < 0.0001

To understand the role of Claudin5 in esophageal cancer, we conducted a comprehensive analysis of its expression in both public databases and our collected clinical samples. Utilizing the TIMER2.0 (http://timer.cistrome.org/), UALCAN (https://ualcan.path.uab.edu/), and GEPIA (http://gepia.cancer-pku.cn/) portals, we examined the mRNA expression level of *CLDN5* across various cancer types (Fig. 1a). Interestingly, our analysis revealed higher *CLDN5* expression in primary esophageal cancer tissue compared to adjacent normal tissue, indicative of its potential significance in ESCC (Fig. 1b). Despite the lack of statistical significance in the UALCAN analysis, likely due to the limited sample size of the normal tissue group (only 11 cases) compared to the primary tissue group (184 cases), our findings suggested a trend towards lower Claudin5 expression in squamous cell carcinoma compared to adenocarcinoma within the ESCA subtype (Fig. 1c).

In China, squamous cell carcinoma is the primary histological subtype of ESCA. To further investigate Claudin5 expression in ESCC, we analyzed protein expression levels using IHC in ESCC tissue samples from 123 patients. Our analysis revealed a significant decrease in Claudin5 protein expression in ESCC tissues compared to adjacent normal tissues (Fig. 1d–f). This finding aligns with the observations from bioinformatics analysis using public databases. Additionally, we found a relatively lower Claudin5 expression in patients with grade 3 tumors compared to grades 1–2 (Fig. 1g). Furthermore, a significant association was observed between low Claudin5 expression and advanced T stage as well as node metastasis (Fig. 1h, i).

Taken together, these findings demonstrate the downregulation of Claudin5 in ESCC tissues and indicate a potential association between Claudin5 expression and tumor progression.

### Claudin5 knockdown promotes proliferation, invasion and migration of ESCC cell in vitro and in vivo

**Fig. 2:**
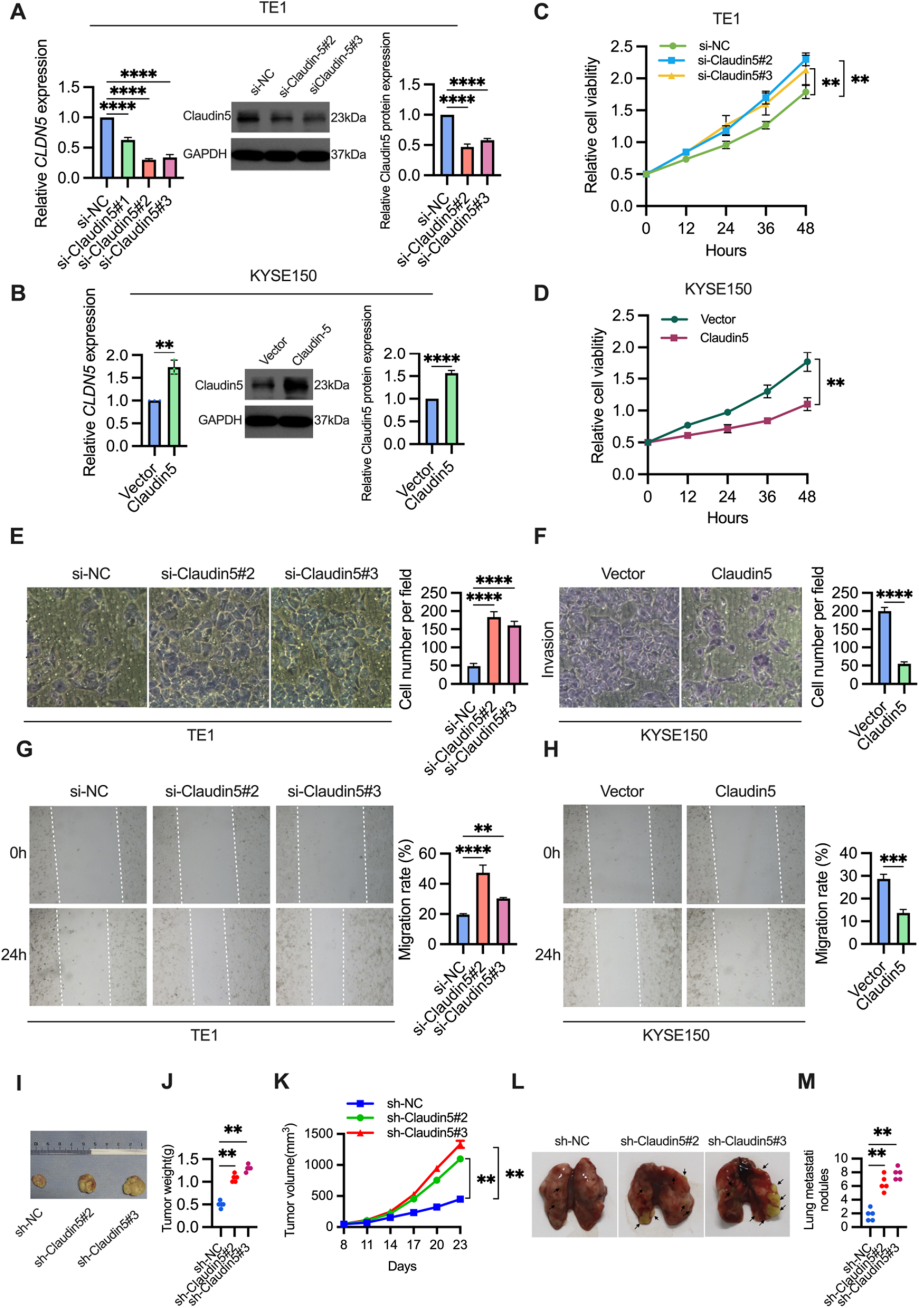
Claudin5 knockdown promotes proliferation, invasion and migration of ESCC cells in vitro and in vivo. **A**, **B** Claudin5 expression levels were assessed in TE1 cells transfected with Claudin5 siRNA (**A**), and in KYSE150 cells transfectsed with Claudin5 (**B**) using qRT-PCR and western blotting. GAPDH served as the loading control. **C**, **D** The effects of Claudin5 knockdown (**C**) or overexpression (**D**) on the ESCC cells proliferation were evaluated by CCK-8 assay. **E**, **F** The effects of Claudin5 knockdown (**E**) or overexpression (**F**) on ESCC cells invasion were evaluated by transwell invasion assay (magnification, × 40). **G**, **H** The effects of Claudin5 knockdown (**G**) or overexpression (**H**) on ESCC cells migration were evaluated by wound healing assay (magnification, × 200). **I** Representative data of tumors in nude mice bearing TE1 cells. **J** Tumor weight of xenograft mouse tumors. **K** Tumor volumes of xenograft mouse tumors. **L**, **M** Claudin5 knockdown resulted in increased lung metastasis, shown by the elevated number of lung nodules. The statistical difference was assessed with one-way ANOVA followed by Dunnett tests in **A**, **C**, **E**, **G**,** J**,** K** and** M**; and the two-tailed unpaired Student *t* test in **B**, **D, F** and **H**. Error bars show the SD from three independent experiments. **p < 0.01, ***p < 0.001, ****p < 0.0001

To elucidate the role of Claudin5 in ESCC cells, we employed siRNA to knock it down in TE1 cells and plasmid overexpression in KYSE150 cells based on the Claudin5 expression level in ESCC cells from the DepMap database (https://depmap.org/portal/). The efficiency of knockdown and overexpression was confirmed by qRT-PCR and western blotting (Fig. 2a, b). Cell proliferation assays demonstrated that TE1 cells with reduced Claudin5 expression exhibited significantly higher proliferation activity compared to the control group (Fig. 2c). Conversely, overexpression of Claudin5 using plasmid transfection inhibited the proliferation activity of KYSE150 cells (Fig. 2d).

We then evaluated whether Claudin5 affected the invasion and migration abilities of ESCC cells. Transwell invasion assay showed that Claudin5 knockdown promoted the invasion capacity of TE1 cell (Fig. 2e). Wound healing assay showed that Claudin5 knockdown also promoted the migration ability of TE1 cells (Fig. 2g). Overexpresssion of Claudin5 reduced the invasion and migration ability of KYSE150 cells (Fig. 2f, h).

To further validate the effects of Claudin5 knockdown, in vivo xenograft and lung metastasis experiments were conducted. Knockdown of Claudin5 resulted in increased tumor weight and larger tumor volume (Fig. 2i–k). Additionally, the xenograft model with Claudin5 knockdown exhibited a significant reduction (over 50%) in the number of lung metastatic nodules (Fig. 2l, m), indicating a pronounced promotion of lung metastasis.

Overall, these results highlight the role of Claudin5 knockdown in enhancing the malignant phenotypes of ESCC cells.

### Downregulation of Claudin5 reduced radiosensitivity of ESCC cells

**Fig. 3:**
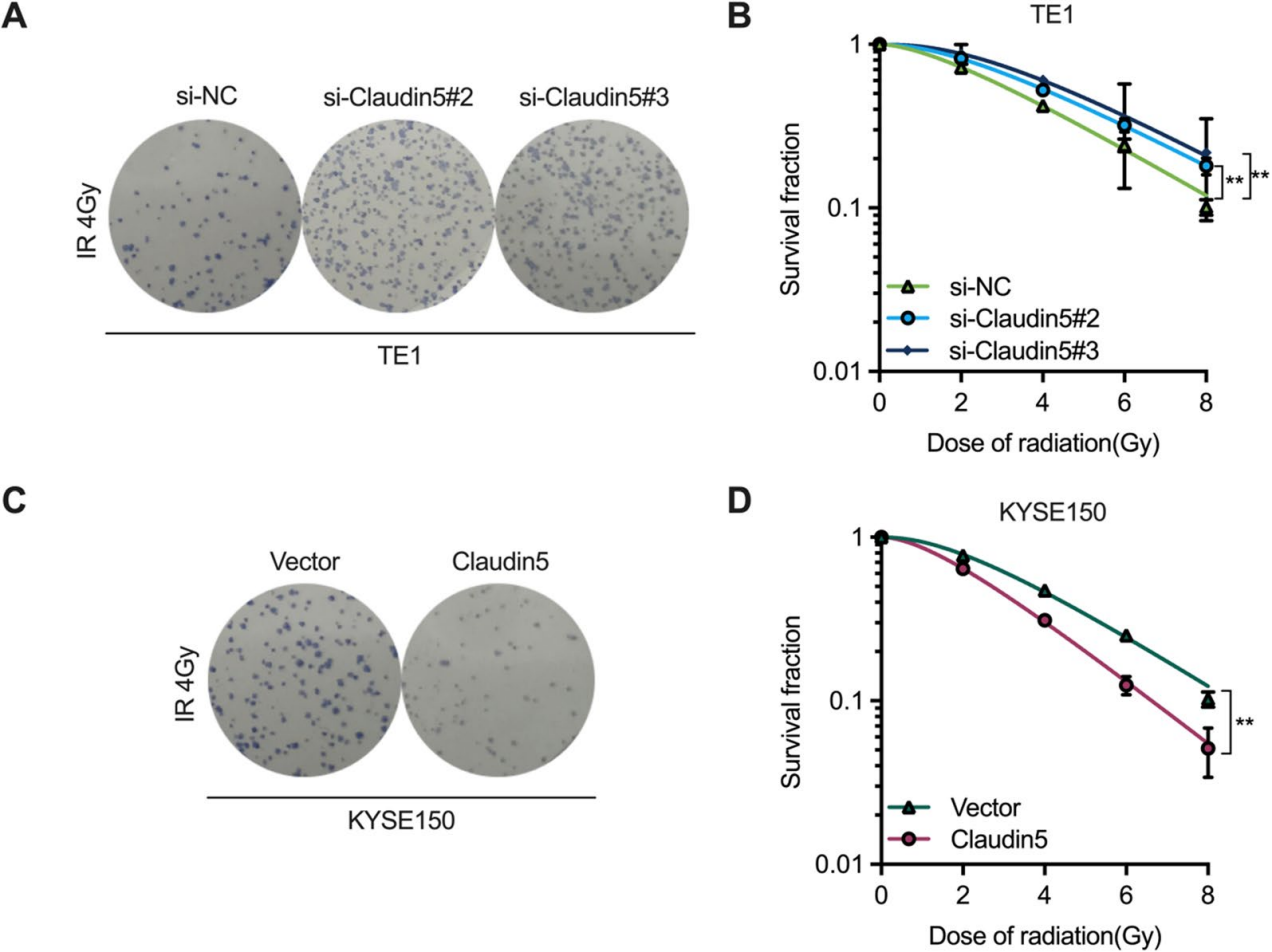
Downregulation of Claudin5 reduced radiosensitivity of ESCC cells. Clonogenic survival assay was used to evaluate the effects of Claudin5 on ESCC cells radiosensitivity. **A**, **C** Representative images of colony formation after irradiation. **B**, **D** Survival curves of ESCC cells treated with irradiation. The survival curves were plotted using GraphPad Prism Version 9.5.1 according to the single-hit multitarget model (SF = 1 − (1 − e^ [− kD])^N). Associated radiobiological parameters (*D*_0_, *D*_*q*_, *N*, and SF2) were calculated and shown in Table 1. The statistical difference was assessed with one-way ANOVA followed by Dunnett tests in **B**; and the two-tailed unpaired Student *t* test in **D**. Error bars show the SD from three independent experiments. **p < 0.01

Radiotherapy is the primary treatment for advanced ESCC patients. However, cancer cell resistance presents a significant obstacle to patient survival [3]. Therefore, it is crucial to investigate the molecular mechanism of radioresistance. As Claudin5 has been shown to inhibit the malignant behavior of ESCC cells, we hypothesized that it may also be involved in regulating radioresistance. To confirm this hypothesis, we conducted clonogenic survival assay on TE1 and KYSE150 cells, as previously described [17, 18]. Knockdown of Claudin5 increased TE1 cell colony formation after irradiation, reducing their radiosensitivity (Fig. 3a) as seen in the survival curve (Fig. 3b). Conversely, overexpression of Claudin5 inhibited KYSE150 cell colony formation and increased radiosensitivity (Fig. 3c), supported by the survival curve (Fig. 3d). Table 1 shows the detailed radiobiological parameters of the cells. These results indicate that downregulation of Claudin5 can promote ESCC cell survival after irradiation and inhibit radiosensitivity.

**Table 1:** Radiobiological parameters of ESCC cells (associated with Fig. 3)

Cell lines	Group	*D*_0_ (Gy)	*D*_*q*_ (Gy)	*N*	SF2 (%)
TE1	si-NC	2.98 ± 0.12	2.39 ± 0.12	1.80 ± 0.14	72.11 ± 1.60
	si-Claudin5#2	3.29 ± 0.14^a^^,^*	3.82 ± 0.14^a^^,^*	2.16 ± 0.16^a^^,^*	82.12 ± 2.50^a^^,^*
	si-Claudin5#3	3.20 ± 0.13^b,^*	5.53 ± 0.23^b,^*	2.73 ± 0.15^b,^*	87.60 ± 1.20^b,^*
KYSE150	Vector	2.76 ± 0.12	3.62 ± 0.20	2.31 ± 0.15	77.40 ± 1.80
	Claudin5	2.25 ± 0.16^c,^*	2.13 ± 0.17^c,^*	1.95 ± 0.19^c,^*	64.01 ± 1.20^c,^*

Radiobiological parameters (*D*_0_, *D*_*q*_, *N*, and SF2) were calculated from the single-hit multitarget model (SF = 1 − (1 − e^ [− kD])^N) using GraphPad Prism Version 9.5.1 software. Data represent three independent experiments (mean ± SD)

*D*_0_, final slope; *D*_*q*_, quasi-threshold dose; *N*, extrapolation number; SF2, survival fraction of 2 Gy

*p < 0.05, two-tailed *t*-test, unpaired

^a^Si-Claudin5#2 versus si-NC

^b^Si-Claudin5#3 versus si-NC

^c^Claudin5 versus Vector

### Supppression of Claudin5 promotes autophagy

**Fig. 4:**
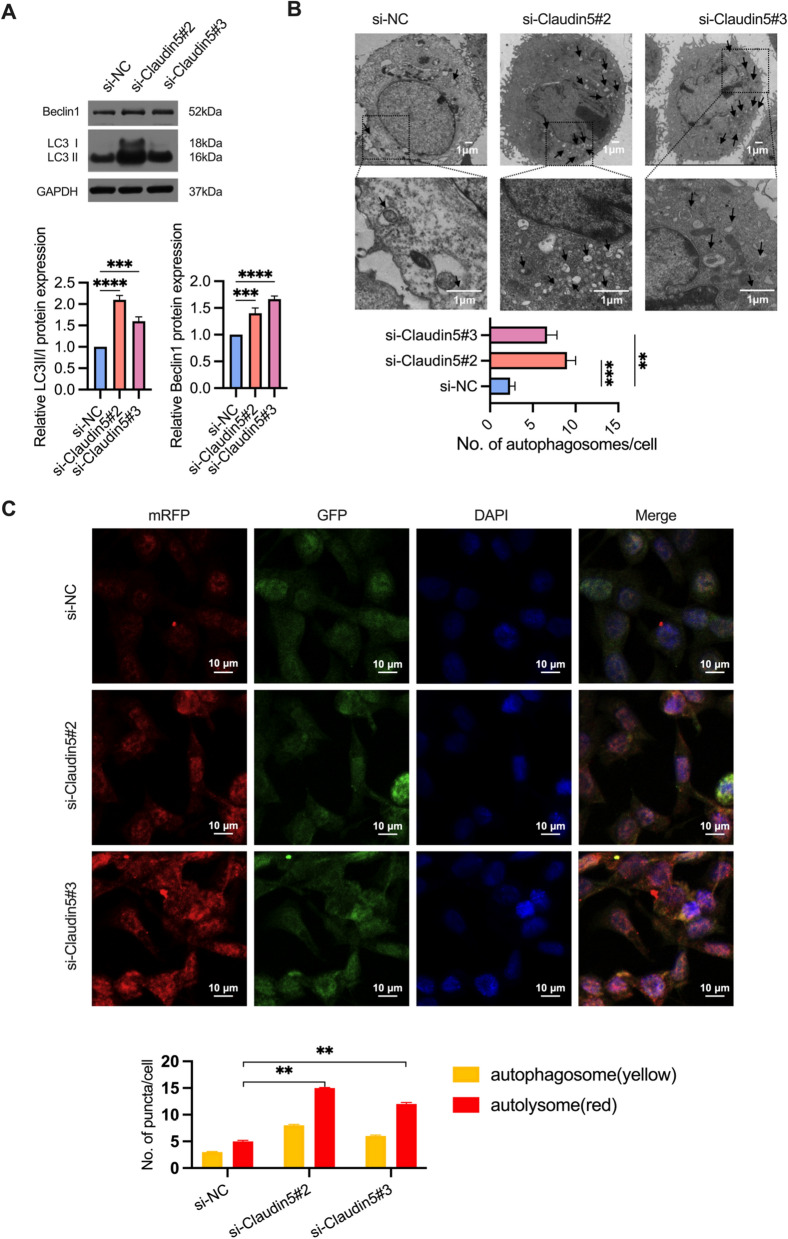
Claudin5 knockdown promotes autophagy in ESCC cells. **A** Western blotting was performed to detect LC3 I, LC3 II and Beclin1 protein expression in TE1 cells transfected with Claudin5 siRNA. GAPDH served as the loading control. **B** transmission electron microscopy (TEM) images of autophagosomes in TE1 cells transfected with Claudin5 siRNA. **C** mRFP-GFP-LC3-transfected TE1 cells were treated with Claudin5 siRNA, and laser confocal fluorescence microscopy was used to analyze autophagosomes and autolysosomes. Merged image shows yellow puncta for autophagosomes and red puncta for autolysosomes. The puncta were analyzed by ImageJ software. The statistical difference was assessed with one-way ANOVA followed by Dunnett tests in **A**, **B** and **C**. Error bars show the SD from three independent experiments. **p < 0.01, ***p < 0.001, ****p < 0.0001

In our previous study, we discovered that autophagy promotes cell survival under irradiation and increases radioresistance [17]. To further investigate the correlation between Claudin5 and autophagy in ESCC cells, we evaluated the impact of Claudin5 on autophagy. By western blotting [20], we detected the expression of the autophagy formation marker LC3 II/I and observed that knockdown of Claudin5 in TE1 cells increased the LC3 II/I protein levels (Fig. 4a). We also used transmission electron microscopy (TEM) [20] to test the effect of Claudin5 on intracellular autophagosomes. TEM images showed that Claudin5-knockdown TE1 cells had more autophagosomes than control cells (Fig. 4b). Additionally, we tracked the formation and degradation of autophagy using GFP-RFP-LC3 [20] and found that knockdown of Claudin5 improved autophagy flux in TE1 cells (Fig. 4c). Interestingly, we found that Beclin1, the key regulatory factor in autophagy [8], was upregulated by the downregulation of Claudin5 in TE1 cells (Fig. 4a). In summary, our findings suggest that Claudin5 knockdown promotes autophagy activation in ESCC cells.

### Beclin1-mediated autophagy drives malignant progression and radioresistance in Claudin5-downregulated esophageal squamous cell carcinoma

**Fig. 5:**
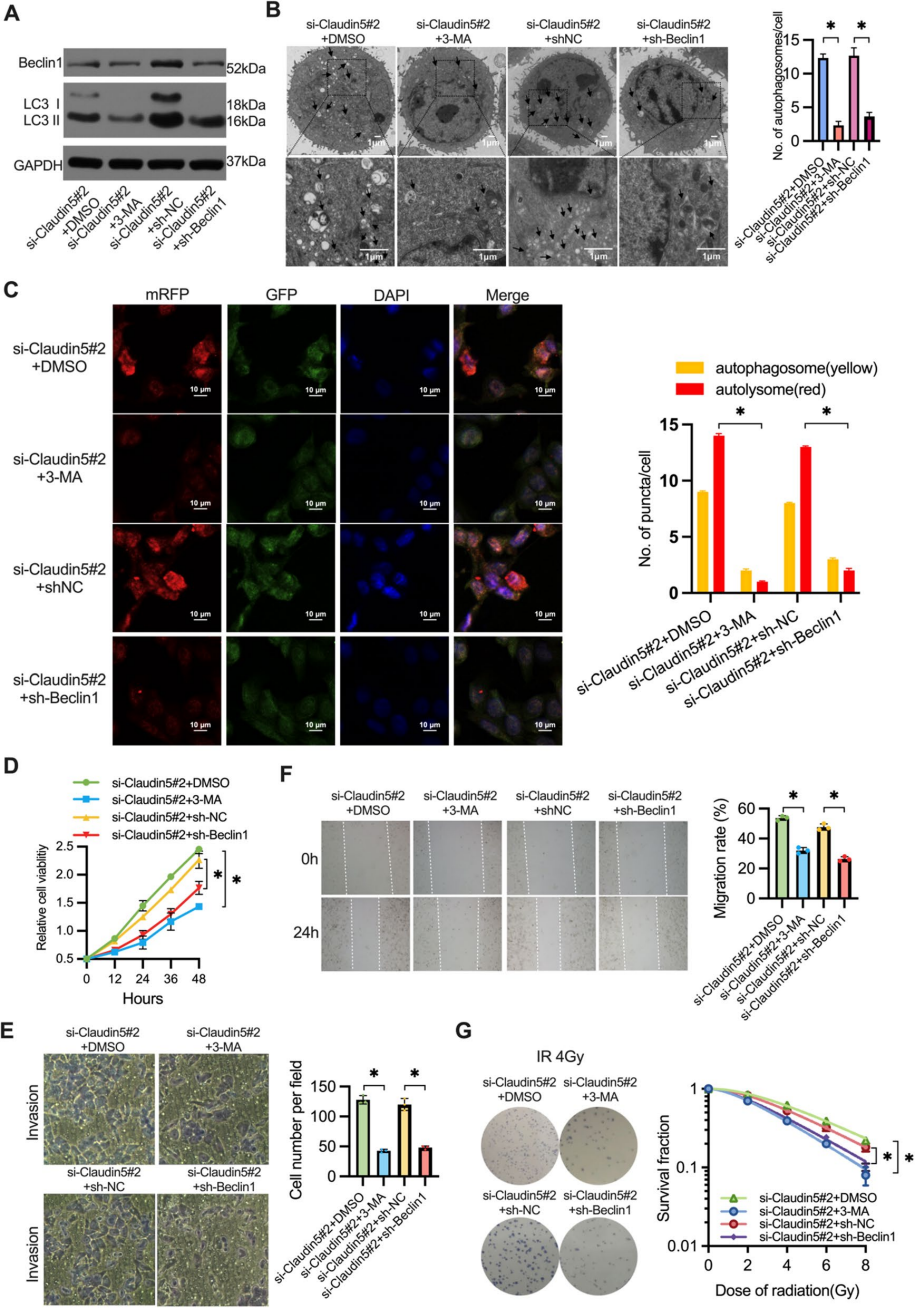
Downregulation of Claudin5 promotes malignant progression and radioresistance through Beclin1-mediated autophagy. **A** Western blotting was performed to detect LC3 I, LC3 II and Beclin1 protein expression in TE1 cells transfected with Claudin5 siRNA, Beclin1 shRNA or treated with 3-MA for 4 h. GAPDH served as the loading control. **B** TEM images of autophagosomes in TE1 cells transfected with Claudin5 siRNA, Beclin1 shRNA or treated with 3-MA for 4 h. **C** Laser confocal fluorescence microscopy was used to analyze autophagosomes and autolysosomes in TE1 cells transfected with Claudin5 siRNA, Beclin1 shRNA or treated with 3-MA for 4 h. Merged image shows yellow puncta for autophagosomes and red puncta for autolysosomes. The puncta were analyzed by ImageJ software. **D**–**E** The ability of proliferation (**D**), invasion (**E**, magnification, × 40) and migration (**F**, magnification, × 200) in TE1 cells transfected with Claudin5 siRNA, Beclin1 shRNA or treated with 3-MA for 4 h was assessed by CCK-8 assay, transwell invasion assay and wound healing assay. **G** The radiosensitivity in TE1 cells transfected with Claudin5 siRNA, Beclin1 shRNA or treated with 3-MA for 4 h was assessed by clonogenic survival assay. The survival curves were plotted using GraphPad Prism Version 9.5.1 according to the single-hit multitarget model (SF = 1 − (1 − e^ [− kD])^N). Associated radiobiological parameters (*D*_0_, *D*_*q*_, *N*, and SF2) were calculated and shown in Table 2. The statistical difference was assessed with the two-tailed unpaired Student *t* test in **B**, **C**, **D**, **E**, **F** and **G**. Error bars show the SD from three independent experiments. *p < 0.05

Based on the promotion of autophagy and Beclin1 expression observed in ESCC cells with Claudin5 downregulation, we conducted further investigations to explore the role of autophagy and Beclin1 in the malignant phenotype induced by Claudin5 knockdown in ESCC. Autophagy was inhibited using 3-MA (an common molecule inhibitor of early phases of autophagy), in Claudin5 knockdown TE1 cells to elucidate its impact on the malignant phenotype. The successful inhibition of autophagy was confirmed through various analyses (Fig. 5a–c). The inhibition of autophagy led to a partial reduction in the proliferative activity of Claudin5 knockdown cells (Fig. 5d). Similarly, autophagy inhibition partially attenuated the invasive capacity (Fig. 5e) and migration activity (Fig. 5f) of Claudin5 knockdown cells. Moreover, the clonogenic assay demonstrated that autophagy inhibition partially reversed the radioresistance observed in Claudin5 knockdown cells (Fig. 5g), emphasizing the involvement of autophagy in the malignant phenotype driven by Claudin5 knockdown in ESCC.

**Table 2:** Radiobiological parameters of ESCC cells (associated with Fig. 5g)

Cell lines	Group	*D*_0_ (Gy)	*D*_*q*_ (Gy)	*N*	SF2 (%)
TE1	si-Claudin5#2 + DMSO	3.58 ± 0.17	4.85 ± 0.14	2.35 ± 0.22	85.00 ± 1.20
	si-Claudin5#2 + 3-MA	2.69 ± 0.20^a^^,^*	2.39 ± 0.24^a^^,^*	1.89 ± 0.12^a^^,^*	70.00 ± 1.8^a^^,^*
	si-Claudin5#2 + sh-NC	3.29 ± 0.16	3.82 ± 0.21	2.16 ± 0.15	82.12 ± 2.50
	si-Claudin5#2 + sh-Beclin1	2.98 ± 0.13^b,^*	2.39 ± 0.14^b,^*	1.80 ± 0.13^b,^*	72.11 ± 1.60^b,^*

Radiobiological parameters (*D*_0_, *D*_*q*_, *N*, and SF2) were calculated from the single-hit multitarget model (SF = 1 − (1 − e^ [− kD]) ^N) using GraphPad Prism Version 9.5.1 software. Data represent three independent experiments (mean ± SD)

*D*_0_, final slope; *D*_*q*_, quasi-threshold dose; *N*, extrapolation number; SF2, survival fraction of 2 Gy

*p < 0.05, two-tailed *t*-test, unpaired

^a^Si-Claudin5#2 + DMSO versus si-Claudin5#2 + 3-MA

^b^Si-Claudin5#2 + sh-NC versus si-Claudin5#2 + sh-Beclin1

To further investigate the role of Beclin1 in the Claudin5 knockdown-induced malignant phenotype, Beclin1 was knocked down in Claudin5-downregulated TE1 cells using shRNA (Fig. 5a). Similar to the effects of 3-MA treatment, Beclin1 knockdown significantly inhibited autophagy. Western blotting analysis confirmed the downregulation of LC3 II/I protein levels in Claudin5-downregulated TE1 cells upon Beclin1 knockdown (Fig. 5a). TEM and autophagy flux assays further supported the suppression of autophagy levels in Claudin5-downregulated TE1 cells following Beclin1 knockdown (Fig. 5b, c).

Furthermore, we examined the involvement of Beclin1 in the oncogenic effects of Claudin5 downregulation. Knockdown of Beclin1 partially attenuated the proliferation-promoting effect of Claudin5 downregulation in TE1 cells (Fig. 5d). Additionally, Beclin1 knockdown rescued the impact of Claudin5 on cell invasion (Fig. 5e) and migration (Fig. 5f). Notably, Claudin5 downregulation enhanced the resistance of TE1 cells to irradiation, and Beclin1 knockdown partially reversed this radioresistance (Fig. 5g). In summary, these findings strongly support the conclusion that Beclin1-mediated autophagy plays a pivotal role in the promotion of malignant progression, radioresistance, and activation of autophagy in ESCC cells with Claudin5 downregulation.

### Low Claudin5 expression is associated with reduced radiotherapy response and poor patient prognosis

**Fig. 6:**
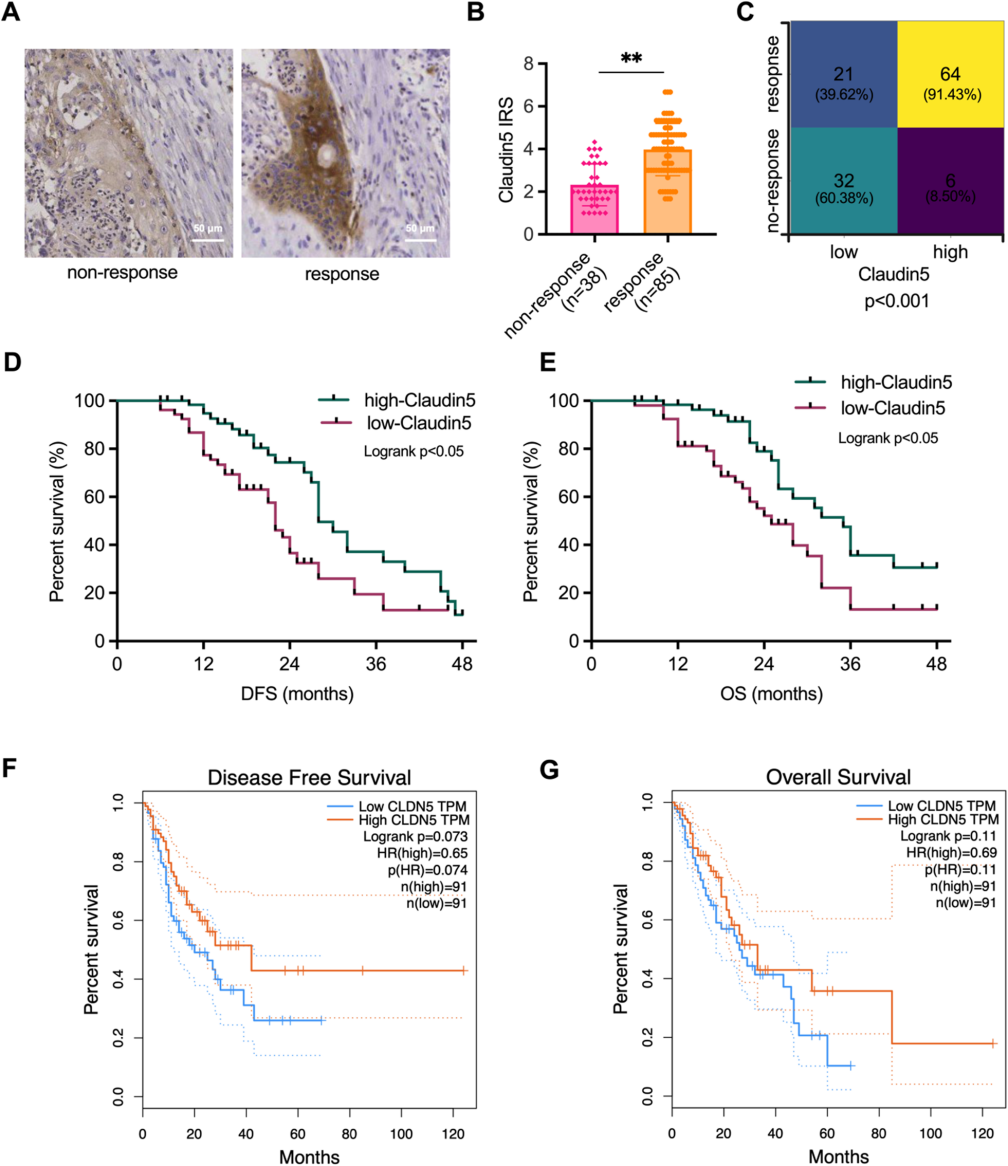
Lower Claudin5 correlates with unfavorable patient outcomes and decreased radiotherapy response. **A** Representative IHC staining of Claudin5 protein in collected clinical ESCC samples from non-response (n = 38) and response (n = 85) patients to radiotherapy. **B** IRS of Claudin5 protein in clinical ESCC samples. **C** Claudin5 protein expression in tumors and radiotherapy response rate in clinical ESCC samples. **D**, **E** Kaplan–Meier analyses of disease-free survival (DFS) and overall survival (OS) for collected clinical ESCC patients with high or low tumor Claudin5 expression. **F**, **G** Kaplan–Meier analyses of DFS (**F**) and OS (**G**) for ESCA patients with high or low tumor Claudin5 expression using TCGA database. The statistical difference was assessed with the unpaired nonparametric Mann–Whitney U test in **B**; Chi-square test in **C**; and the Logrank test for **D**, **E**, **F** and **G**; Error bars show the SD from three independent experiments. **p < 0.01

Lacking a marker to identify which subgroup of ESCC patients would benefit from radiotherapy, many patients have received unnecessary radiotherapy [3]. To address this issue, we evaluated the association between Claudin5 expression and the response to radiotherapy in 123 clinical ESCC patients. The ESCC patients were classified into either a radiotherapy response group or a non-response group based on their tumor size as determined by imaging after radiotherapy. Lower expression of Claudin5 was observed in the non-response group, as shown by IHC staining (Fig. 6a, b). Moreover, low Claudin5 expression was associated with a poor response to radiotherapy (Fig. 6c), indicating that patients with low Claudin5 levels in their tumor were less likely to benefit from radiotherapy.

After demonstrating the role of Claudin5 in tumor cell progression, we further evaluated its relationship with patient survival. Specifically, low Claudin5 expression correlated with poorer disease-free survival (DFS) and overall survival (OS) (Fig. 6d, e). We also verified this the finding in 182 esophageal cancer patients from the TCGA database. The TCGA analysis showed a consistent trend, where patients with low *CLDN5* expression exhibited worse DFS and OS compared to those with high *CLDN5* expression (Fig. 6f, g). It is worth noting that the lack of statistical significance may be due to the mixture of squamous cell carcinoma and adenocarcinoma cases in the TCGA dataset. Collectively, these data suggest a correlation between Claudin5 expression levels and patients prognosis, highlighting the potential use of Claudin5 as a predictive biomarker in ESCC.

## Discussion

ESCC is a prevalent malignancy with poor response to radiotherapy, and the investigation of its molecular mechanisms and treatment strategies is urgently needed. In this study, we have uncovered a novel function of Claudin5 in regulating ESCC cell progression and enhancing radiosensitivity. Our findings indicate that downregulation of Claudin5 promotes malignant progression and radioresistance through Beclin1-mediated autophagy in ESCC. Furthermore, we have demonstrated a positive correlation between Claudin5 expression levels and radiotherapy response, as well as patient prognosis, highlighting the potential of Claudin5 as a predictive biomarker in ESCC.

Despite numerous studies exploring the relationship between claudins and tumors, the role of Claudin5 (*CLDN5*) in tumorigenesis remains largely unexplored [6]. Our study provides novel insights into the role of Claudin5 in ESCC. We found that Claudin5 expression is suppressed in ESCC tumors, consistent with previous findings in other cancer types such as lung cancer [21, 22], breast cancer [23], and colon cancer [24]. Moreover, our functional experiments revealed the specific effects of Claudin5 on cells proliferation, migration, invasion, and radiosensitivity in ESCC. These findings contribute to the existing knowledge on Claudin5's role in cancer progression. Additionally, our clinical analysis demonstrated an association between low Claudin5 expression and poor prognosis in ESCC patients, consistent with previous findings in human breast cancer by Yang [23]. Notably, we identified a previously unreported positive correlation between Claudin5 expression and radiotherapy response in ESCC. This novel finding has clinical relevance and highlights the potential of Claudin5 as a predictive biomarker for radiotherapy response in ESCC patients.

Autophagy is a fundamental metabolic process responsible for the degradation and recovery of intracellular substances [25, 26]. Tight junctions, including Claudin5, play a critical role in maintaining the epithelial barrier, polarity, and signal transmission [27, 28]. Our study revealed that Claudin5 inhibits autophagy in ESCC cells, providing new insights into the regulatory role of Claudin5 in this process. Although our findings are novel for Claudin5, studies on other members of the claudin family partially support our results. For instance, Claudin1 has been shown to induce drug resistance mediated by autophagy in non-small cell lung cancer by ULK1 phosphorylation [29], and it acts as an autophagy stimulant, accelerating the degradation of SQSTM1/p62 [30]. Additionally, our previous studies have demonstrated that irradiation triggers protective autophagy, promoting cell irradiation resistance [17]. Consistent with these findings, our study reveals that autophagy mediates the effect of Claudin5 on cell malignant phenotype and radiosensitivity. Furthermore, we found that Beclin1 is a downstream target of Claudin5 involved in regulating autophagy. Previous studies has reported the role of Beclin1 in cancer radiosensitivity. For example, Beclin1/autophagy signaling mediates Sox2-promoted chemoresistance, proliferation, stemness, migration, and invasion in colorectal cancer cells [31], and IL-6 activates autophagy via the IL-6/JAK2/BECN1 pathway, promoting chemotherapy resistance in colorectal cancer [32]. Our results align with these reports and underscore the significance of Beclin1-mediated autophagy in regulating the radiosensitivity of ESCC cells.

While our study provides valuable insights, it also has several limitations that should be acknowledged. Firstly, confirming the clinical significance of Claudin5 as a prognostic biomarker would benefit from a larger clinical cohort. Secondly, the limited number of available normal adjacent tissue samples may have reduced the statistical power of our analysis and introduced potential bias in the interpretation of the results. Future studies should aim to collect larger and more representative sample sizes to strengthen our findings. Thirdly, although we elucidated the downstream regulatory mechanisms of Claudin5 involving Beclin1, exploring the upstream regulatory mechanisms of Claudin5 is warranted.

## Conclusions

In summary, our study identifies Claudin5 as a novel suppressor gene of ESCC and elucidates its mechanism of action in inhibiting cell migration and invasion through Becalin-mediated autophagy. Our findings shed light on the interplay between Claudin5 and autophagy in ESCC cells and highlights the potential of Claudin5 as a predictive biomarker and therapeutic target for ESCC, given its correlation with poor radiotherapy response and clinical outcome. Based on our results and our previous study that observed the activation of autophagy in radioresistant ESCC and showed that inhibiting autophagy improved radiosensitivity, we suggest that future research could explore the use of Claudin5 as a potential therapeutic target in combination with radiotherapy for ESCC patients. Additionally, further investigation is needed to uncover the precise molecular mechanisms by which Claudin5 regulates autophagy and to evaluate the feasibility of targeting the Claudin5/autophagy axis as a treatment strategy for ESCC.

## Supplementary Information

**Additional file 1. Table S1**: Sequences of siRNA. **Table S2**: Sequences of shRNAs. **Table S3**: Primer sequences used for qPCR.

## Data Availability

The datasets used during the current study are available from the corresponding author on reasonable request.

## References

[1] Sung H, Ferlay J, Siegel RL, Laversanne M, Soerjomataram I, Jemal A (2021). Global cancer statistics 2020: GLOBOCAN estimates of incidence and mortality worldwide for 36 cancers in 185 countries. CA Cancer J Clin.

[2] Li S, Chen H, Man J, Zhang T, Yin X, He Q (2021). Changing trends in the disease burden of esophageal cancer in China from 1990 to 2017 and its predicted level in 25 years. Cancer Med.

[3] Luo Y, Mao Q, Wang X, Yu J, Li M (2018). Radiotherapy for esophageal carcinoma: dose, response and survival. Cancer Manag Res.

[4] Mineta K, Yamamoto Y, Yamazaki Y, Tanaka H, Tada Y, Saito K (2011). Predicted expansion of the claudin multigene family. FEBS Lett.

[5] Tabariès S, Siegel PM (2017). The role of claudins in cancer metastasis. Oncogene.

[6] Morin PJ (2005). Claudin proteins in human cancer: promising new targets for diagnosis and therapy. Can Res.

[7] Wang D-W, Zhang W-H, Danil G, Yang K, Hu J-K (2022). The role and mechanism of claudins in cancer. Front Oncol.

[8] Glick D, Barth S, Macleod KF (2010). Autophagy: cellular and molecular mechanisms. J Pathol.

[9] Chen N, Debnath J (2010). Autophagy and tumorigenesis. FEBS Lett.

[10] Yun CW, Lee SH (2018). The roles of autophagy in cancer. Int J Mol Sci.

[11] Kroemer G, Mariño G, Levine B (2010). Autophagy and the integrated stress response. Mol Cell.

[12] Shang L, Chen S, Du F, Li S, Zhao L, Wang X (2011). Nutrient starvation elicits an acute autophagic response mediated by Ulk1 dephosphorylation and its subsequent dissociation from AMPK. Proc Natl Acad Sci.

[13] Parzych KR, Klionsky DJ (2014). An overview of autophagy: morphology, mechanism, and regulation. Antioxid Redox Signal.

[14] Chaachouay H, Ohneseit P, Toulany M, Kehlbach R, Multhoff G, Rodemann HP (2011). Autophagy contributes to resistance of tumor cells to ionizing radiation. Radiother Oncol.

[15] Tam SY, Wu VWC, Law HKW (2017). Influence of autophagy on the efficacy of radiotherapy. Radiat Oncol.

[16] Taylor MA, Das BC, Ray SK (2018). Targeting autophagy for combating chemoresistance and radioresistance in glioblastoma. Apoptosis.

[17] Ma H, Zheng S, Zhang X, Gong T, Lv X, Fu S (2019). High mobility group box 1 promotes radioresistance in esophageal squamous cell carcinoma cell lines by modulating autophagy. Cell Death Dis.

[18] Huang S, Li X, Chen X, Che S, Chen W, Zhang X (2013). Inhibition of microRNA-21 increases radiosensitivity of esophageal cancer cells through phosphatase and tensin homolog deleted on chromosome 10 activation. Dis Esophagus.

[19] Remmele W, Stegner HE (1987). Recommendation for uniform definition of an immunoreactive score (IRS) for immunohistochemical estrogen receptor detection (ER-ICA) in breast cancer tissue. Pathologe.

[20] Klionsky DJ, Abdel-Aziz AK, Abdelfatah S, Abdellatif M, Abdoli A, Abel S (2021). Guidelines for the use and interpretation of assay for monitoring autophagy. Autophagy.

[21] Akizuki R, Shimobaba S, Matsunaga T, Endo S, Ikari A (2017). Claudin-5, -7, and -18 suppress proliferation mediated by inhibition of phosphorylation of Akt in human lung squamous cell carcinoma. Biochim Biophys Acta Mol Cell Res.

[22] Yang R, Zhou Y, Du C, Wu Y (2020). Bioinformatics analysis of differentially expressed genes in tumor and paracancerous tissues of patients with lung adenocarcinoma. J Thorac Dis.

[23] Yang G, Jian L, Chen Q (2021). Comprehensive analysis of expression and prognostic value of the claudin family in human breast cancer. Aging (Albany).

[24] Bujko M, Kober P, Mikula M, Ligaj M, Ostrowski J, Siedlecki JA (2015). Expression changes of cell-cell adhesion-related genes in colorectal tumors. Oncol Lett.

[25] Feng Y, He D, Yao Z, Klionsky DJ (2014). The machinery of macroautophagy. Cell Res.

[26] Li X, He S, Ma B (2020). Autophagy and autophagy-related proteins in cancer. Mol Cancer.

[27] Ma SC, Li Q, Peng JY, Zhouwen JL, Diao JF, Niu JX (2017). Claudin-5 regulates blood-brain barrier permeability by modifying brain microvascular endothelial cell proliferation, migration, and adhesion to prevent lung cancer metastasis. CNS Neurosci Ther.

[28] Yang Z, Huang C, Wu Y, Chen B, Zhang W, Zhang J (2019). Autophagy protects the blood-brain barrier through regulating the dynamic of Claudin-5 in short-term starvation. Front Physiol.

[29] Zhao Z, Li J, Jiang Y, Xu W, Li X, Jing W (2017). CLDN1 increases drug resistance of non-small cell lung cancer by activating autophagy via up-regulation of ULK1 phosphorylation. Med Sci Monit Int Med J Exp Clin Res.

[30] Kim J, Choi S, Kim JO, Kim KK (2018). Autophagy-mediated upregulation of cytoplasmic claudin 1 stimulates the degradation of SQSTM1/p62 under starvation. Biochem Biophys Res Commun.

[31] Zhu Y, Huang S, Chen S, Chen J, Wang Z, Wang Y (2021). SOX2 promotes chemoresistance, cancer stem cells properties, and epithelial-mesenchymal transition by beta-catenin and Beclin1/autophagy signaling in colorectal cancer. Cell Death Dis.

[32] Hu F, Song D, Yan Y, Huang C, Shen C, Lan J (2021). IL-6 regulates autophagy and chemotherapy resistance by promoting BECN1 phosphorylation. Nat Commun.

