# Supplementary material for: Downregulation of Claudin5 promotes malignant progression and radioresistance through Beclin1-mediated autophagy in esophageal squamous cell carcinoma
Source: J Transl Med. 2023 Jun 11;21:379. doi: 10.1186/s12967-023-04248-7 (PMC10257837; doi:10.1186/s12967-023-04248-7)
Supplement: Supplementary file 1 — Additional file 1. Table S1: Sequences of siRNA. Table S2: Sequences of shRNAs. Table S3: Primer sequences used for qPCR. [file 12967_2023_4248_MOESM1_ESM.docx]

| Name | Sense(5’to3’) | Antisense(5’to3’) |
| --- | --- | --- |
| si-Claudin5#1 | CCACAACAUCGUGACGGCGCA | UGCGCCGUCACGAUGUUGUGG |
| si-Claudin5#2 | CUCUGCUGGUUCGCCAACAUU | AAUGUUGGCGAACCAGCAGAG |
| si-Claudin5#3 | GCGACUACGACAAGAAGAATT | UUCUUCUUGUCGUAGUCGCTT |
| si-NC | UUCUCCGAACGUGUCACGUTT | ACGUGACACGUUCGGAGAATT |

**Supplemental Table S1**. Sequences of siRNA.

**Supplementary Table S2.** Sequences of shRNAs.

| Name | sequence | lentiviral vector |
| --- | --- | --- |
| sh-Claudin5#1 | CCGGCTCTGCTGGTTCGCCAACATTCTCGAGAATGTTGGCGAACCAGCAGAGTTTTTT | pLKO.1-CMV-copGFP-PURO |
| sh-Claudin5#2 | CCGGGCACATGCAGTGCAAAGTGTACTCGAGTACACTTTGCACTGCATGTGCTTTTTT | pLKO.1-CMV-copGFP-PURO |
| sh-Claudin5#3 | CCGGGTTCGTTGCGCTCTTCGTGACCTCGAGGTCACGAAGAGCGCAACGAACTTTTTT | pLKO.1-CMV-copGFP-PURO |
| sh-Beclin1#1 | CCGGCCCGTGGAATGGAATGAGATTCTCGAGAATCTCATTCCATTCCACGGGTTTTTT | pLKO.1-CMV-copGFP-PURO |
| sh-Beclin1#2 | CCGGGCCAGGATGATGTCCACAGAACTCGAGTTCTGTGGACATCATCCTGGCTTTTTT | pLKO.1-CMV-copGFP-PURO |
| sh-Beclin1#3 | CCGGCTCAAGTTCATGCTGACGAATCTCGAGATTCGTCAGCATGAACTTGAGTTTTTT | pLKO.1-CMV-copGFP-PURO |
| sh-NC | NC for TsingKe | pLKO.1-CMV-copGFP-PURO |

**Supplemental Table S3**. Primer sequences used for qPCR.

| Gene | Sequence(5'-3') | |
| --- | --- | --- |
| *CLDN5* | forward primer | CTGACCTTCTCCTGCCACTAG |
|  | reverse primer | GAAGCGAAATCCTCAGTCTGACA |
| *BECN1* | forward primer | CTGAGGGATGGAAGGGTCTAAGA |
|  | reverse primer | CACGGTCCAGGATCTTGAAACTC |
| *GAPDH* | forward primer | TGGACCTGACCTGCCGTCTAGAAA |
|  | reverse primer | GTGGGTGTCGCTGTTGAAGTCAGA |
